# Reforming pedagogy in biotechnology pharmaceutics education: a narrative review of emerging teaching strategies

**DOI:** 10.3389/fpsyg.2026.1915540

**Published:** 2026-09-22

**Authors:** Xiao-huan Liu, Fei Liu, Na Xu, Tao Wang

**Affiliations:** 1 School of Biological Science, Jining Medical University, Jining, China; 2 Key Laboratory of Standardization of Chinese Medicine (Chengdu University of Traditional Chinese Medicine), Ministry of Education, Lab for Innovation & Effective Uses of Chinese Drug Germplasm Resources, School of Pharmacy, Chengdu University of Traditional Chinese Medicine, Chengdu, Sichuan, China

**Keywords:** biotechnology pharmaceutics, flipped classroom, industry-academia collaboration, problem-based learning, teaching reform

## Abstract

The rapid expansion of the biopharmaceutical industry, driven by monoclonal antibodies, cell and gene therapies, and mRNA vaccines, demands a workforce equipped with interdisciplinary knowledge, practical competence, and critical thinking. Traditional lecture-based instruction in Biotechnology Pharmaceutics usually falls short in cultivating these attributes. This narrative review, drawing on a structured synthesis of recent literature (2015–2026) and the authors’ 10 years of front-line teaching experience, addresses the following research question: What pedagogical innovations hold the greatest promise for transforming biopharmaceutics education, and what are their respective evidence bases, implementation challenges, and pathways to scalability? It examines problem-based and case-based learning, flipped classrooms, virtual simulation, industry-academia integration, and curriculum ideological and ethical education. The review evaluates the strengths, implementation challenges, and evidence of effectiveness for each innovation, identifying three key dimensions of analysis: pedagogical effectiveness, implementation feasibility, and scalability. The findings indicate that while short-term cognitive and motivational gains are well-documented across all five strategies, longitudinal evidence and systemic adoption frameworks remain critically underdeveloped. The review is limited by the predominantly short-term, single-institution nature of the available evidence base and the scarcity of studies examining implementation fidelity across diverse institutional contexts. Accordingly, the paper highlights areas requiring further faculty development, institutional support, and proposes a concrete five-component framework for future longitudinal research to address these evidence gaps. It concludes that an integrated and student-centered ecosystem combining multiple active pedagogies, authentic experiential learning, and robust digital tools represents the most promising path forward, contingent on sustained institutional commitment and systematic scaling strategies.

## 1 Introduction

Biotechnology pharmaceutics stands as a quintessentially translational discipline, one that does not merely sit at the crossroads of disparate fields but actively fuses them into a coherent whole (Hoang Anh et al., 2020; Ghavami and Łos, 2025). It bridges molecular biology and genetic engineering with bioprocess engineering, quality regulation, and clinical application. By 2022, biopharmaceuticals accounted for nearly 40% of the total pharmaceutical market (Walsh and Walsh, 2022), which are mainly driven by monoclonal antibodies, CAR-T cell therapies, and mRNA vaccines. Two paradigm shifts (including precision medicine and continuous manufacturing) are particularly disruptive to established educational models (Subbiah, 2023; Domokos et al., 2021). Against this backdrop, the inadequacies of traditional pedagogical structures become starkly visible (How et al., 2025; Stonier and Jones, 2025). For decades, biopharmaceutics education has relied on a teacher-centered architecture of fragmented lectures (allocating discrete sessions to topics such as cell line development, chromatography, and viral clearance) interspersed with confirmatory laboratory exercises that require little more than protocol execution. These exercises typically require students to execute pre-written, step-by-step protocols—a “cookbook” approach that insulates learners from the cognitive demands of authentic process development. Moreover, the assessment systems reinforce this superficial engagement, predominantly relying on summative written examinations that reward recall of isolated facts rather than integrative reasoning.

This cognitive gap is not a student deficiency but a predictable outcome of instructional design. Traditional lectures cultivate passive learning habits and fail to exercise the integrative problem-solving and risk-assessment skills essential for careers in bioprocess development, technology transfer, and quality assurance. The challenge is compounded by the interdisciplinary nature of modern biopharmaceutical production, where a single decision (choosing a chemically defined medium over a serum-containing one) ripples across downstream purification, analytical characterization, and regulatory filing. Recognizing these deficiencies, a global wave of pedagogical experimentation has emerged, grounded in a robust evidence-based consensus that active learning strategies outperform traditional lecturing. Landmark meta-analyses demonstrate that active engagement reduces failure rates, enhances conceptual understanding (Yannier et al., 2021), and narrows achievement gaps among diverse student populations (Theobald et al., 2020). Parallel systematic reviews in engineering education confirm that problem-based and collaborative learning strengthen the higher-order competencies that biopharmaceutical science demands (Chen et al., 2020a).

In response, reform movements have reshaped how we educate the next generation of biopharmaceutical scientists. Problem-based and case-based learning immerse students in authentic, ill-structured problems; flipped and blended classrooms prioritize active problem-solving over passive listening; high-fidelity virtual simulations democratize access to expensive or hazardous equipment with significant gains in achievement and motivation (Santos and Prudente, 2022); and deep industry–academia partnerships bring current good manufacturing practice into the curriculum. These technical reforms are increasingly woven together with curriculum-embedded ethical education, which cultivate the holistic professional judgment that an industry dealing with life-saving therapies demands.

The review is guided by the following research question: What pedagogical innovations hold the greatest promise for transforming biopharmaceutics education, and what are their respective evidence bases, implementation challenges, and pathways to scalability? The objective of this review is to critically synthesize the literature on five emerging pedagogical strategies (PBL, CBL, flipped classroom, virtual simulation, industry-academia integration, and ethical education) with respect to three analytical dimensions: (a) pedagogical effectiveness (demonstrated learning outcomes), (b) implementation feasibility (barriers and facilitators), and (c) scalability (potential for systemic adoption). We do not advance a formal hypothesis as this is a narrative review; however, we critically evaluate the proposition, implicit in much of the reform literature, that each innovation independently improves learning outcomes. Our analysis suggests that this proposition requires qualification: we argue that these strategies are most effective when implemented as an integrated, coherent ecosystem rather than as isolated interventions.

While existing literature has examined individual pedagogical innovations in isolation, the present review offers three original contributions that go beyond prior syntheses. First, we propose an integrated, student-centered teaching ecosystem that synthesizes six interdependent modules (including active teaching, practical training, industry docking, value education, assessment alignment, and institutional support) into a coherent framework for curricular transformation. Second, we develop a concrete, five-component framework for future longitudinal research to address the critical evidence gap regarding long-term graduate outcomes, an issue acknowledged but rarely operationalized in the literature. Third, we provide practical, evidence-based strategies for resource-constrained institutions (Section 3.4) and a systematic roadmap for scaling pilot innovations to systemic adoption, addressing two persistent challenges that prior reviews have largely overlooked.

## 2 The shortcomings of conventional pedagogy

For decades, the educational architecture of biotechnology pharmaceutics has been constructed around a transmission model of instruction. The archetypal curriculum partitions the discipline into a sequence of self-contained lecture modules: upstream cell culture and bioreactor operations are taught in isolation from downstream chromatography and filtration; formulation science appears as a separate entity detached from the analytical methods that characterize it; and regulatory affairs are often relegated to a capstone overview that students perceive as a mere recitation of ICH guideline categories. This fragmentation is not accidental but structural. Faculty members, themselves specialists in narrow domains, deliver meticulously prepared slide decks that emphasize declarative knowledge (e.g., the classification of mammalian cell lines, the kinetics of Michaelis–Menten, the steps of a viral inactivation procedure) while seldom illuminating the web of interdependencies that a practicing bioprocess engineer must navigate daily. The result, as I have witnessed semester after semester, is a cohort that can enumerate the four pillars of Quality by Design but cannot construct a design space for a critical quality attribute when given a real data set.

The lecture-centric paradigm cultivates, and indeed rewards, a passive learning disposition. Students become skilled at transcribing, memorizing, and reproducing information, yet they rarely engage in the cognitive struggle that forges durable, transferable understanding. Recent work in cognitive psychology and discipline-based education research has clarified the mechanism underlying this phenomenon: students in traditional lectures frequently report a higher sense of fluency and perceived learning, yet objective measures demonstrate that they learn significantly less than their peers in actively taught sections (Deslauriers et al., 2019). This “illusion of learning” is particularly hazardous in biopharmaceutics, where the ability to reason from first principles under conditions of ambiguity (for instance, diagnosing the root cause of an unexpected turbidity increase during a scale-up run) depends not on recognition of memorized facts but on flexible, conditionalized knowledge structures that passive instruction rarely builds.

The laboratory component, which should serve as the crucible for procedural and diagnostic skill development, has too often degenerated into a ritualistic exercise of protocol execution. In a typical confirmatory laboratory session, students pipette pre-prepared solutions, follow step-by-step chromatography scripts, and record observations whose expected values are already known. Decision-making is virtually absent; students are not asked to select an appropriate resin from a panel of candidates, to justify a wash buffer pH based on protein isoelectric point, or to determine whether an observed peak represents a genuine product variant or a column artefact. When laboratory work is stripped of inquiry, it ceases to function as a vehicle for scientific reasoning and becomes what has been termed a “cookbook” activity (Hofstein and Lunetta, 2004). Recent best-practice guidelines for instructional laboratories stress that prescribed and confirmatory exercises fail to cultivate the modelling, troubleshooting, and design competencies that constitute the core rationale for hands-on science education (Smith and Holmes, 2021). The implications for biopharmaceutics are profound: a student who has never designed a purification experiment, made a methodological error, and corrected it is ill-prepared for the iterative and failure-laden reality of process development.

The assessment systems that overlay these instructional practices further entrench surface-level learning. The dominant instrument remains the summative and closed-book written examination, typically composed of multiple-choice items and short-answer questions that probe factual recall: “List the three stages of viral clearance validation,” “State the typical pCO₂ setpoint for a CHO cell bioreactor,” or “Define the acronym PAT.” Such assessments are undeniably reliable for ranking students and are economical to administer in large-enrolment classes. However, they are profoundly misaligned with the integrated competencies that define professional practice. An engineer writing a deviation report, a scientist drafting a comparability protocol, or a regulator evaluating a biosimilar application must synthesize knowledge from upstream, downstream, analytical, and quality domains simultaneously. Conventional examinations do not sample this integrative capacity, nor do they assess communication, teamwork, or ethical reasoning. A recent systematic review of assessment literature confirms that when evaluation emphasizes reproduction over application, students might adopt a strategic and surface approach to learning that inhibits deep understanding and long-term retention (Goss, 2022).

The cumulative consequence of fragmented lectures, protocol-driven laboratories, and recall-oriented examinations is a generation of graduates who (despite solid grade-point averages) exhibit a striking inability to transfer their knowledge to the messy and multi-variable problems of industry. A major synthesis of active-learning research, published in a leading interdisciplinary journal, concluded that “hands-on” must be coupled with “minds-on” engagement for robust conceptual gains. The passive and lecture-dominated instruction consistently yields inferior outcomes across STEM disciplines (Yannier et al., 2021). This finding is reinforced by large-scale studies showing that active pedagogies not only elevate overall performance but also narrow persistent achievement gaps for students from underrepresented groups (Theobald et al., 2020). In the specific domain of bioprocess engineering education, these broad findings have catalyzed a recognition that incremental adjustments (adding a single case study or a guest lecture) are insufficient. A growing chorus of educators calls for a fundamental reimagining of curricula, assessment, and faculty roles to align educational practice with the cognitive demands of the modern biopharmaceutical industry (Henkel et al., 2015).

It is this collective diagnosis that has propelled the search for alternative pedagogies (Table 1). The principle is now clear: to educate a biopharmaceutical scientist who can design, troubleshoot, and innovate, we must replace the passive, fragmented, and surface-oriented model with one that is active, integrated, and contextually authentic. The following sections of this review examine the specific innovations, problem-based learning, flipped classrooms, virtual simulation, industry integration, and ethical education, that constitute the vanguard of this transformation.

**Table 1:** Gaps between conventional pedagogy and the competency demands of modern biopharmaceutical practice.

Conventional pedagogy feature	Manifested deficiency	Required competency
Fragmented, module-based lectures (upstream/downstream/regulatory taught separately)	Inability to integrate knowledge across disciplinary boundaries	Interdisciplinary reasoning and systems thinking
Passive lecture-dominated instruction	Illusion of learning; weak transfer to novel problems	Active problem-solving under ambiguity
Confirmatory “cookbook” laboratory exercises	Lack of troubleshooting and experimental design skills	Iterative process development and root-cause analysis
Recall-oriented summative written examinations	Surface learning; inability to apply knowledge to authentic scenarios	Integrated application, communication, and ethical reasoning

## 3 Key methodological innovations

### 3.1 Problem-based and case-based learning (PBL/CBL)

Among the active pedagogies advanced to remedy the deficiencies of conventional biopharmaceutics instruction, problem-based learning (PBL) and case-based learning (CBL) have emerged as particularly powerful vehicles for cultivating the integrative decision-making mindset the industry demands. While they share a family resemblance, PBL and CBL rest on distinct instructional logics that can be deployed synergistically.

In PBL, a complex and ill-structured problem is presented to students before they have received prerequisite content, which serves as the catalyst for knowledge acquisition rather than its application. A representative trigger in biopharmaceutics might ask students to develop a viral clearance validation strategy for a novel bispecific antibody, compelling them to identify knowledge gaps (e.g., the viral clearance mechanisms, chromatography, regulatory guidance) and independently acquire the necessary expertise. The instructor acts as a facilitator to guide metacognitive reflection and collaborative sense-making rather than dispensing answers. A recent systematic review of PBL methodologies in engineering education confirms that such approaches yield significant and consistent gains in self-directed learning, knowledge integration, and collaborative problem-solving abilities (Sukackė et al., 2022). At the same time, the PBL literature consistently identifies implementation challenges (including the substantial shift required in faculty role perception, the design of authentic yet achievable problems, and the alignment of assessment with process-oriented learning goals) that must be addressed for sustainable adoption (Chen et al., 2020b) (Figure 1).

**Figure 1:**
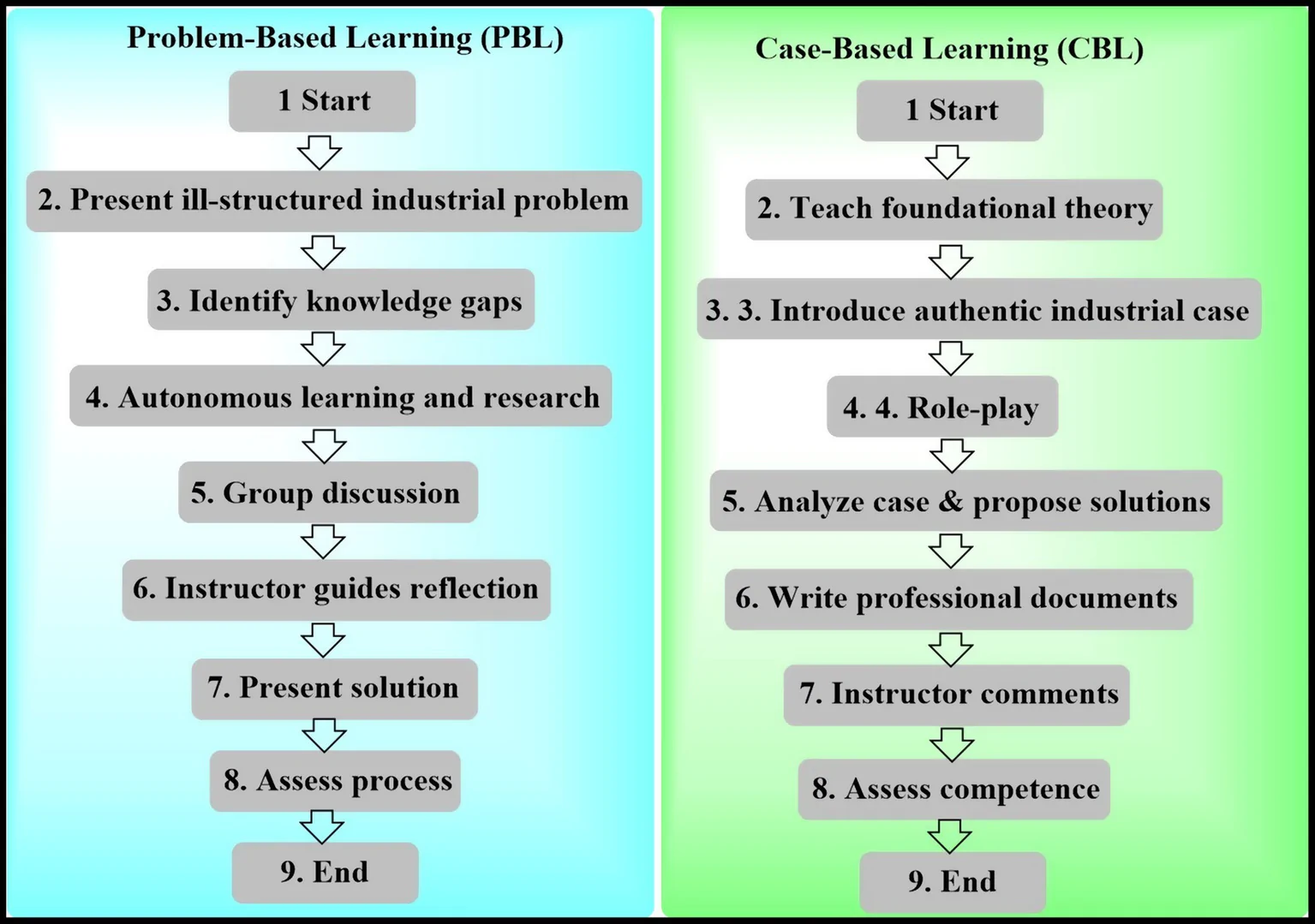
Parallel implementation workflow of PBL and CBL.

CBL, by contrast, employs authentic industrial cases analyzed after foundational concepts have been introduced. A representative case might involve a contamination event in a cGMP facility manufacturing a therapeutic enzyme, complete with environmental monitoring data, personnel movement logs, and preliminary root-cause hypotheses. Students assume the role of the quality assurance investigation team—reconstructing events, prioritizing corrective actions, and drafting sections of a formal deviation report. In Spooner and Kennedy’s elective course, pharmacy students prepared a simulated investigational new drug application and responded to mock agency inquiries. This exercise, when combined with mock regulatory dossier preparation, deepened their learning measurably—enhancing regulatory writing, quality-by-design reasoning, and risk management skills (Spooner and Kennedy, 2017). A comprehensive worldwide review of CBL in medical and healthcare education has similarly concluded that the method enhances clinical reasoning, knowledge application, and professional competence, findings that are directly transferable to the biopharmaceutical sciences (McLean, 2016). More broadly, a review of bioprocess engineering education has argued that PBL and CBL align exceptionally well with the cognitive demands of process development and should form a central pillar of curricular reform (Henkel et al., 2015).

What distinguishes PBL and CBL from conventional instruction is their fidelity to industrial problem-solving. Rather than compartmentalizing upstream, downstream, analytical, and regulatory knowledge, these approaches compel learners to traverse disciplinary boundaries organically. When addressing a viral clearance problem, students simultaneously consider molecular biology, unit operations, assay sensitivity, and regulatory expectations; when investigating a contamination event, they draw on microbiology, facility design, and human factors. Students must formulate hypotheses, interpret ambiguous data, and negotiate solutions under uncertainty—precisely the capabilities that employers consistently report as deficient in new graduates.

Moreover, PBL and CBL address the affective and motivational dimensions that traditional lectures neglect. By anchoring abstract concepts in consequential and real-world scenarios, they foster professional identity and purpose: students are not merely learning about biopharmaceutics; they are practicing it. This identity shift is critical for retaining talent and accelerating the transition from novice to competent professional. Achieving this, however, hinges on careful instructional design: problems must be calibrated to learners’ developmental stages, assessment must evaluate process alongside product, and faculty must be supported in transitioning from information transmitters to learning architects. When these conditions are met, PBL and CBL represent not incremental improvements but a qualitative transformation in biopharmaceutics education (Table 2).

**Table 2:** Comparison of teaching links and roles between PBL and CBL.

Comparison dimension	Problem-based learning (PBL)	Case-based learning (CBL)
Knowledge sequence	Problem first, knowledge learning second	Theoretical teaching first, case application second
Core carrier	Open-ended exploratory problems	Mature real industrial cases
Student main tasks	Find knowledge gaps, autonomous learning and group discussion	Analyze cases, role-play and write professional documents
Teacher role	Facilitator, guide thinking reflection	Instructor, case commentator and skill trainer
Applicable teaching stage	New knowledge learning stage	Knowledge consolidation and professional application stage
Main training goal	Autonomous learning and problem exploration	Knowledge application and professional post competence

### 3.2 Flipped classroom and blended learning

The flipped classroom restructures the learning experience by shifting initial content exposure (traditionally delivered in lectures) to students’ individual preparation time (Figure 2). In biopharmaceutics, foundational topics such as bioreactor design equations, protein folding thermodynamics, and regulatory impurity classifications are delivered through pre-recorded micro-lectures, animated explainers, and curated readings. Class time is thereby repurposed for active and collaborative problem-solving. Students might work through scale-up calculations revealing mass transfer limitations, analyze biophysical data to identify genuine critical quality attributes, or engage in peer instruction to resolve misconceptions. Meta-analyses confirm that flipped classrooms produce moderate but significant improvements in learning outcomes, with greatest gains when in-class time focuses on authentic, problem-centered activities rather than extended mini-lectures (Spaic et al., 2025). In pharmaceutical education, flipped approaches reliably enhance knowledge acquisition and higher-order application skills, which are associated with measurable increases in engagement and participation (Aljaber et al., 2023).

**Figure 2:**
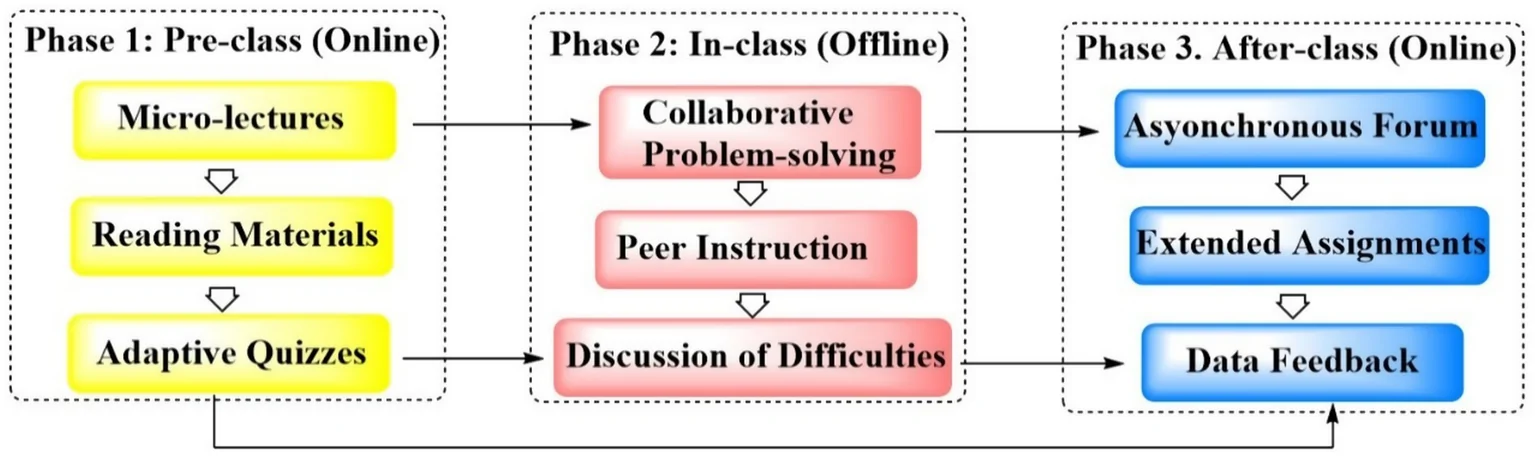
Closed-loop workflow of flipped classroom and blended learning.

Blended learning extends this logic beyond pre-class/in-class alternation to a more integrated ecosystem. Online discussion forums enable asynchronous argumentation on topics such as biosimilar interchangeability; adaptive quizzes provide immediate, individualized feedback, fostering self-regulated preparation; and synchronous virtual workshops connect learners with industry experts for live case consultations. Well-designed blended courses can match or surpass face-to-face effectiveness while offering greater flexibility, with interactive, feedback-rich online components as consistent predictors of positive outcomes (Balakrishnan et al., 2021).

However, success is contingent on digital material quality and instructor facilitation skill. Pre-recorded lectures that merely replicate passive monologues or static online modules fail to sustain motivation and may undermine learning. Poor-quality resources, inadequate instructor presence, and insufficiently integrated design are primary inhibitors of engagement and achievement (Rasheed et al., 2020). Equally critical is the instructor’s transformation from information transmitter to learning architect, which is a role demanding fluency in questioning, formative assessment, group facilitation, and real-time pedagogical decision-making, skills rarely cultivated in doctoral training. In our experience, transitioning to a blended curriculum required substantial investment in video production, instructional design, and teaching assistant training, which is a considerable but necessary investment to close the gap between passive, fragmented learning and the integrative competence the biopharmaceutical industry demands.

### 3.3 Virtual simulation and digital laboratories

A authentic, hands-on training in biotechnology pharmaceutics has long been constrained by three practical barriers: the prohibitive cost of pilot-scale bioreactors and chromatography skids, biosafety risks inherent in handling live viruses and pathogenic microorganisms, and severely restricted access to cGMP suites that cannot be casually repurposed for teaching (Figure 3). These constraints have historically limited students to observing industrial operations through glass windows or brief plant tours—experiences that inform but do not immerse. Virtual simulation platforms have fundamentally altered this landscape. Learners can now operate interactive, digitally twinned bioreactors that respond to feeding strategy changes in real time, explore chromatography resin selection effects on purity and yield through *in silico* experimentation, and navigate multi-step cleanroom entry and aseptic gowning procedures in fully immersive 3D environments. A systematic review confirms that such simulations are most effective when they require active exploration and decision-making rather than passive viewing, and when thoughtfully blended with hands-on activities (Radianti et al., 2020).

**Figure 3:**
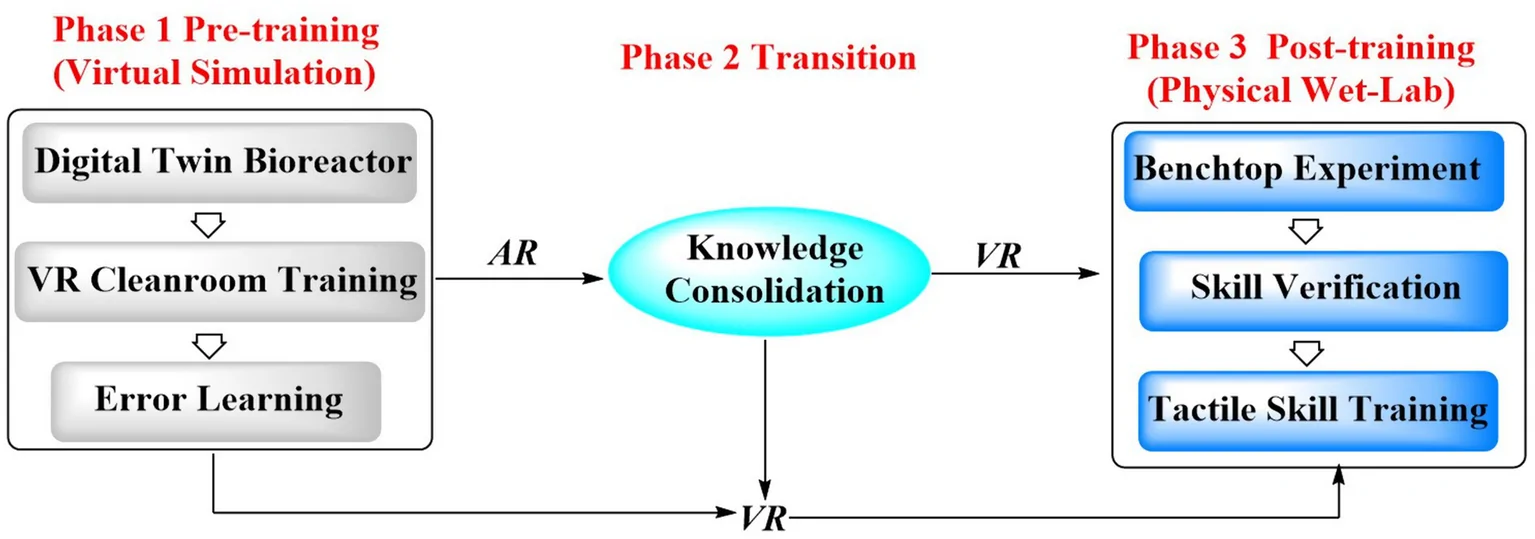
Hybrid framework of virtual simulation and physical laboratories.

Controlled studies in bioprocess engineering education reinforce this conclusion. A hybrid instructional model—virtual upstream processing experiments followed by focused benchtop sessions—enhances procedural competence and safety awareness more effectively than conventional laboratory exercises alone (Sari et al., 2019). The virtual phase allows students to learn from errors that would be too costly or dangerous in a physical lab, while subsequent wet-lab experience consolidates tactile skills and appreciation for biological variability. A comprehensive meta-analysis further found that well-designed virtual laboratories produce significant gains in achievement and motivation, often matching or exceeding outcomes of physical laboratories.

The strategic value of digital laboratories was magnified during the COVID-19 pandemic, when campus closures severed students from physical facilities. Educators globally adopted virtual simulation platforms to maintain practical education continuity. Gamage et al. (2020) concluded that virtual labs, paired with remote guidance, were indispensable for preserving experimental reasoning skills. This forced experiment revealed the durability and scalability of digital tools, accelerating their integration into mainstream curricula.

Emerging technologies promise to push experiential learning further. Augmented reality (AR) overlays digital information onto physical equipment, which enables students to practice assembly, column packing, or manifold setup with real-time guidance and error alerts, reducing costly mistakes and reinforcing correct procedures. Virtual reality (VR) is being developed for aseptic technique training, immersing students in simulated cleanrooms where they must maintain airflow awareness, transfer materials without compromising sterility, and respond to detected breaches. Early evidence from clinical skills education indicates that these immersive technologies is capable of significantly improving procedural knowledge transfer and self-efficacy (Moro et al., 2021). Their extension into biopharmaceutics promises to democratize access to high-fidelity experiential learning, enabling any student, regardless of institutional resources, to practice and master the skills demanded by regulated biopharmaceutical manufacturing (Table 3).

**Table 3:** Advantages and limitations of virtual simulation laboratory and traditional physical laboratory.

Evaluation index	Virtual simulation laboratory	Traditional physical laboratory
Equipment and operation cost	Low; one-time development + routine maintenance	High; expensive pilot equipment and consumables
Biosafety and operational risk	No virus, microbial and operational safety hazards	Existing biological safety and operation risks
Fault tolerance	Allow repeated wrong operations, support error learning	Wrong operation may cause equipment damage or safety accidents
Training content	Focus on process logic, parameter adjustment and system cognition	Focus on hands-on operation, manual skills and actual sample processing
Scale and popularization	Unlimited number of students, remote access available	Restricted by laboratory space and equipment quantity
Limitations	Lack of tactile experience of real operation	Limited training scenarios, high experimental failure cost

### 3.4 Practical approaches for resource-constrained institutions

While the benefits of virtual simulations are well-established, many institutions (particularly those in resource-constrained settings) face significant barriers to implementation, including high software licensing costs, limited IT infrastructure, and lack of technical expertise. However, practical, scalable approaches can overcome these barriers. We synthesize four evidence-based strategies.

First, leveraging free and open-source virtual lab platforms offers an immediate, no-cost entry point. Platforms such as ChemCompute provide free web-based access to computational chemistry software; RemoteLabz (Flauzac et al., 2026) offers network simulation capabilities; and the All-Science Sims project provides 234 interactive simulations across 14 subjects, running entirely in a web browser. These platforms require only internet access and standard browsers, which make them accessible to institutions with minimal IT infrastructure.

Second, adopting hybrid virtual-physical teaching models maximizes learning outcomes while minimizing equipment costs. Recent research on instrumental analysis education demonstrated that a virtual-physical hybrid model represents “the most efficient and economical solution for teaching scenarios with limited resources” (Fei et al., 2026). The virtual phase allows students to learn from errors without consuming expensive reagents or risking equipment damage, while the subsequent physical phase consolidates tactile skills. This approach has been validated in analytical chemistry and genetics education, where combining face-to-face sessions with online simulations effectively taught gene editing and DNA sequencing (Vedova et al., 2025).

Third, utilizing open educational resources (OERs) and low-cost institution-developed solutions provides a sustainable pathway. During the COVID-19 pandemic, educators developed gamified virtual microbiology labs using PowerPoint-based platforms at no cost, and these demonstrate that sophisticated simulations can be created with commonly available software (Ge et al., 2026). Low-cost VR laboratories have been successfully implemented in Latin American nursing education, enhancing engagement in resource-limited contexts (Castillo et al., 2025). In engineering education, 3D-printed equipment combined with virtual simulations offers another low-cost alternative (Waheed et al., 2025). AI-enhanced virtual laboratories have also demonstrated an 80.9% cost reduction while providing unlimited experimental access, without compromising pedagogical effectiveness (Ge et al., 2026).

Fourth, establishing cross-institutional partnerships and resource-sharing consortia enables institutions to pool resources and share costs. Collaborative programming models have been successfully implemented to scale up pharmacy education in resource-limited countries. Partnerships between institutions in different resource settings (such as the collaboration between the University of Zimbabwe and Monash University to develop virtual pharmacy software) demonstrate that resource sharing can overcome individual institutional limitations (Dustman et al., 2021). Similarly, open-source platforms like DOJO for cybersecurity education are freely available and have been successfully used in multiple university courses and workshops (Waheed et al., 2025).

### 3.5 Industry-academia integration

Direct and sustained exposure to industrial realities is not a supplement but an essential catalyst for bridging theory and professional competence. Programs embedding semester-long projects co-supervised by company mentors, structured internships, and curated plant tours immerse students in authentic industrial settings. Through these experiences, students could tackle real manufacturing challenges, such as optimizing cell culture media, troubleshooting lyophilization deviations, or developing process analytical technology roadmaps under operational constraints. These experiences immerse learners in cGMP culture, where documentation, risk assessment, and cross-functional communication are as critical as the underlying science. Students completing industry-based rotations report could markedly deeper the understanding of regulatory operations and manufacturing workflows, with many describing the experience as pivotal in shaping their career trajectories (Lee et al., 2015). Similarly, structured industry can also placement enhance technical competence and professional socialization necessary for seamless workforce transition (Soucie et al., 2023) (Figure 4).

**Figure 4:**
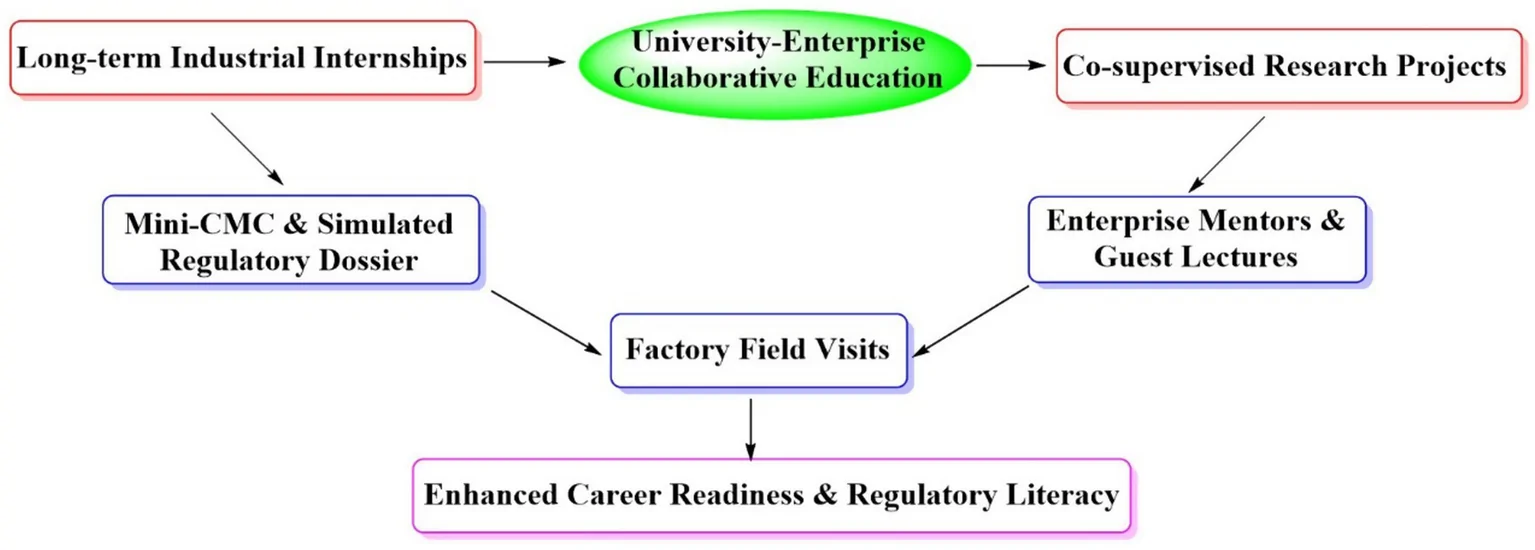
Implementation paths of industry-academia integration.

The collaborative “mini-CMC” project exemplifies this integration. Guided by faculty and industry mentors, student teams construct a complete CMC dossier for a hypothetical biologic—synthesizing cell line history, process description, analytical methods, and container closure into a coherent regulatory narrative. Comparable interventions demonstrate measurable gains in regulatory writing, quality-by-design reasoning, and risk management. For instance, simulated regulatory agency interactions (such as mock Board of Pharmacy meetings and disciplinary hearings) have been shown to enhance pharmacy students’ understanding of regulatory processes and professional decision-making (Parker et al., 2018). Such exercises compel learners to think as regulatory professionals, weighing manufacturing flexibility against filing risk and writing with the precision expected of a Module 3 submission.

In our experience, students returning from co-supervised projects or internships display sharper questioning, heightened motivation, and clearer understanding of career pathways. Industry mentors similarly report that early engagement produces work-ready graduates requiring less professional remediation. Work-integrated learning literature confirms that immersive industry experiences accelerate professional identity formation and career readiness (Wang et al., 2026; Syed et al., 2025), with longitudinal evidence indicating persistent benefits in employment quality and professional agency (Kepple et al., 2025).

Scaling such partnerships remains challenging. Meaningful co-supervision demands reciprocal commitment: academics must cultivate industry relationships, and companies must dedicate mentor-hours away from operational responsibilities. Yet the returns compound for all stakeholders (students, universities, and industry) by producing a talent pipeline that is technically adept, regulatorily literate, and professionally socialized from graduation. As a comprehensive review of bioprocess engineering education argues, sustained industry-academia collaboration is not optional embellishment but a structural necessity for preparing graduates for the interdisciplinary, compliance-driven realities of modern biopharmaceutical manufacturing (Jackson, 2013).

### 3.6 Curriculum ideological and ethical education

Biopharmaceutical innovation touches the most intimate dimensions of human life (from embryonic gene modification to pandemic vaccine allocation) rendering technical competence alone insufficient for professional practice. Recognizing this, educators across diverse contexts have embedded ethical, social, and professional responsibility directly into technical courses, resisting the false dichotomy between “hard” science and “soft” values. In biotechnology pharmaceutics, this integration enriches rather than dilutes scientific content by revealing its societal entanglements. Intentional curriculum design enables instructors to integrate ethical and societal dimensions into technical content without diluting scientific rigor. They might, for example, link drug pricing and equitable access to lyophilization logistics, pair gene-editing ethics with CRISPR-Cas9 molecular biology, discuss biosafety in the context of viral clearance validation, or explore clinical trial transparency through critical appraisal of Phase III data. Such intentional design links technical concepts to moral urgency, which could stimulate moral reasoning, civic awareness, and systemic thinking. This approach aligns with the sociocentric issues (SSI) framework, which demonstrates that engagement with ethically complex dilemmas deepens both scientific understanding and moral sensitivity (Sadler and Zeidler, 2005). Far from political indoctrination, these approaches cultivate holistic professional judgment—the capacity to recognize ethical dimensions, weigh competing values, and act with integrity when regulations are silent. A growing body of research supports this integration: recent analyses argue for a socio-technical orientation in which ethics serves as an integrated lens for technical decisions (Martin et al., 2021), while bioethics education studies confirm that well-designed interventions enhance ethical sensitivity and moral reasoning without sacrificing disciplinary depth (Stratton, 2020). In biopharmaceutics, where a single process decision can determine whether a therapy reaches a patient, this fusion of scientific rigor and ethical reflection is not optional—it is the essence of professional competence (Figure 5, Table 4).

**Figure 5:**
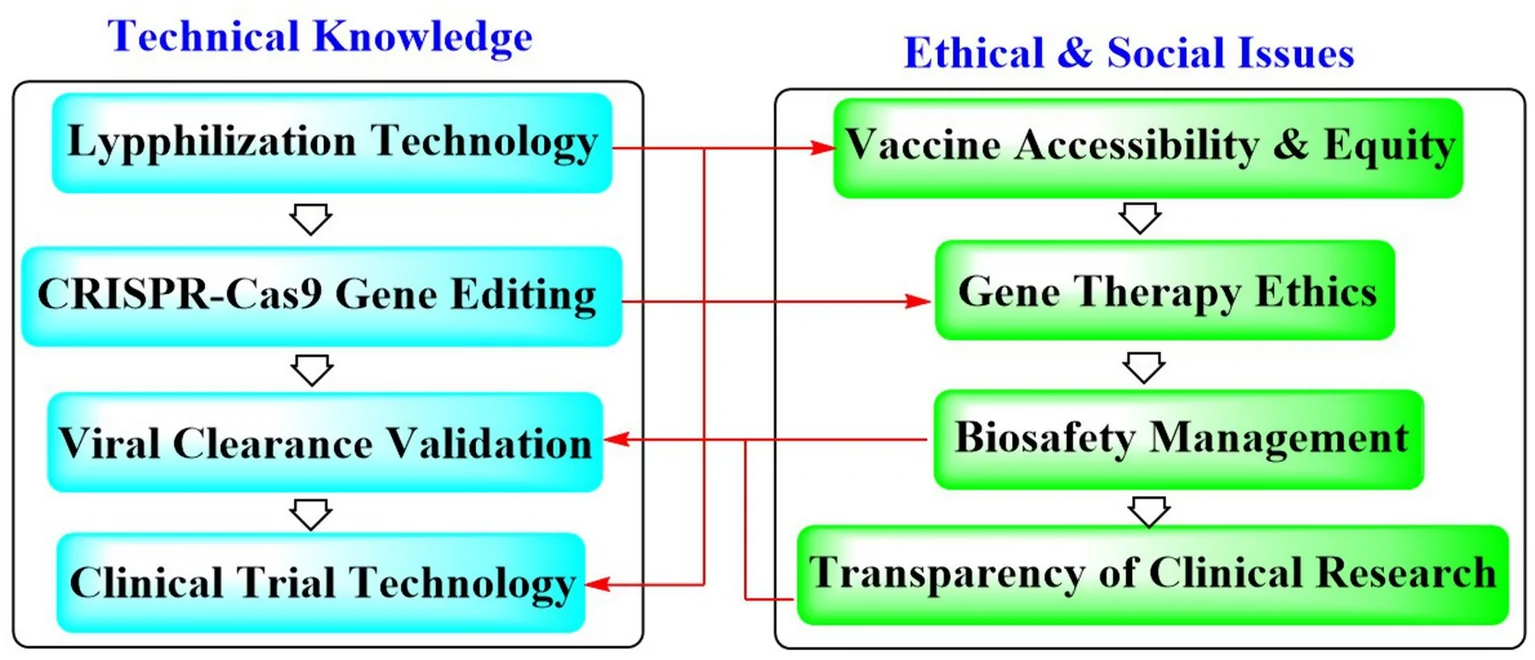
Embedding mode of ideological and ethical education in professional courses.

**Table 4:** Comparative summary of emerging teaching strategies in biotechnology pharmaceutics education.

Strategy	Core principle	Key implementation features	Reported benefits	Major challenges	Evidence level
Problem-based learning (PBL)	Problem drives knowledge acquisition	Ill-structured, open-ended problems; facilitator role; team-based inquiry	Self-directed learning; knowledge integration; collaborative problem-solving	Faculty role shift; authentic problem design; assessment alignment	Moderate to strong (meta-analyses available)
Case-based learning (CBL)	Authentic cases apply knowledge after instruction	Industrial cases; role-play (e.g., QA investigation, regulatory dossier)	Clinical/professional reasoning; knowledge application; professional identity	Case authenticity; instructor facilitation skill	Strong (extensive medical education evidence)
Flipped classroom	Content delivery outside class; active learning in class	Pre-recorded micro-lectures; in-class problem-solving; peer instruction	Enhanced knowledge acquisition; higher engagement; improved application skills	Quality of digital materials; instructor transition; student preparation compliance	Strong (multiple meta-analyses)
Virtual simulation and digital labs	Risk-free, accessible experiential learning	Interactive digital twins; AR/VR cleanroom training; hybrid virtual-physical models	Procedural competence; safety awareness; democratized access	High development cost; technical maintenance; integration with hands-on labs	Moderate to strong (growing evidence)
Industry-academia integration	Authentic professional exposure	Co-supervised projects; internships; mini-CMC dossiers; plant tours	Professional socialization; regulatory literacy; career readiness	Partnership scaling; mentor time commitment; curricular alignment	Moderate (mainly qualitative and case reports)
Curriculum ideological and ethical education	Embedded socio-technical reasoning	SSI framework; ethics woven into technical modules (e.g., vaccine equity, gene editing)	Moral reasoning; civic awareness; systemic thinking	Perceived as “soft” or political; faculty training	Emerging (transferred from SSI literature)

## 4 Assessment alignment and evidence of impact

Teaching reform that does not reach the assessment system is reform halted at the classroom door. If we ask students to reason across disciplines, collaborate in teams, and construct regulatory arguments, yet continue to judge them almost exclusively by recall of isolated facts, we undermine the competencies we claim to value. Forward-looking biopharmaceutics programs have therefore redesigned assessment architectures to align with active and integrative pedagogies. Project portfolios require students to compile a coherent body of work—a development report, purification description, quality risk assessment, and mock regulatory submission. Together, these documents demonstrate sustained, iterative growth across the learning process. Oral defenses before panels including industry practitioners compel students to justify decisions under questioning, mirroring regulatory scrutiny. Peer evaluations develop judgment essential for reviewing batch records and deviation reports. Simulated regulatory inspection, “mock FDA audits”, integrate technical knowledge with communication, diplomacy, and stress management. Objective structured practical examinations (OSPEs) present timed stations (including troubleshooting chromatography scripts, interpreting spectral overlays, drafting responses to out-of-specification results) sampling the breadth of practical reasoning. These approaches embody authentic assessment principles, which research has shown to enhance deep learning, motivation, and knowledge transfer to professional contexts (Table 5) (Villarroel et al., 2018).

**Table 5:** Alignment of assessment methods with learning outcomes in biotechnology pharmaceutics education.

Assessment method	What it measures	Alignment with active/integrative pedagogy	Implementation considerations
Closed-book written examination (traditional)	Factual recall; isolated knowledge fragments	Poor—rewards memorization, not integration	Easy to administer; reliable for ranking
Project portfolio	Sustained, iterative work (e.g., CMC dossier, development report)	Strong—captures process, integration, and growth	Time-intensive to evaluate; requires rubric
Oral defense with industry panel	Justification of decisions under questioning	Strong—simulates regulatory/technical review	Resource-intensive; panel coordination
Peer evaluation	Evaluative judgment; teamwork skills	Moderate—develops reviewer capacity	Requires training; potential bias
Simulated regulatory inspection (mock FDA audit)	Technical knowledge + communication + diplomacy	Strong—high authenticity; stress management	Logistically complex; faculty/industry involvement
Objective structured practical examination (OSPE)	Breadth of practical reasoning (troubleshooting, data interpretation)	Strong—samples multiple competencies	Station design; standardization challenges

The shift toward active and learner-centered education is supported by robust quantitative evidence. Meta-analyses document that active learning significantly increases STEM student performance while reducing failure rates (Freeman et al., 2014). Recent syntheses confirm that “hands-on” engagement must be paired with “minds-on” processing for durable conceptual gains, and that well-designed active pedagogies consistently elevate motivation and satisfaction (Yannier et al., 2021). Within biopharmaceutics and pharmaceutical sciences, qualitative and mixed-methods studies reveal that students gain more than content knowledge from active pedagogies. Those in PBL, flipped, and case-based approaches report marked improvements in critical thinking, interdisciplinary communication, and team collaboration—competencies that conventional lectures rarely foster effectively (Henkel et al., 2015; Cui et al., 2023).

This evidence, however, remains temporally incomplete. The overwhelming majority of studies capture short-term outcomes measured at single-course or semester endpoints. Longitudinal data tracking graduates’ career trajectories, innovation capabilities, and professional resilience over 5–10 years are extraordinarily rare. A systematic review of PBL research across engineering education found that while immediate learning gains are well-documented, few studies follow learners into professional practice to assess durable impacts (Anggraeni et al., 2023). This gap is particularly consequential for biopharmaceutics, where educational reforms are ultimately justified not by examination scores but by the quality, agility, and ethical judgment of professionals who will develop and regulate next-generation biologics. Addressing this deficit represents one of the most urgent priorities for future research in our discipline, which could be achieved through longitudinal, multi-institutional cohort studies linking educational practices to career outcomes.

## 5 Challenges and future directions

The pedagogical innovations surveyed in this review (e.g., PBL, flipped classrooms, virtual simulation, industry co-supervision, and ethics-integrated curricula) hold genuine transformative promise. Yet the gap between promising pilots and sustained system-wide adoption remains dauntingly wide. Translating boutique electives into institutional norms confronts interlocking barriers that are as cultural and structural as they are technical.

### 5.1 Systemic barriers to pedagogical reform

#### 5.1.1 The systemic barriers

The first barrier resides in the professoriate itself. Most faculty in biopharmaceutics programs were trained as research scientists, not educators. They possess deep content expertise but rarely receive formal instruction in learning design, collaborative inquiry facilitation, formative assessment, or the cognitive principles underpinning durable learning. A systematic review confirmed that lack of pedagogical training, combined with concerns about content coverage and classroom control, constitutes a primary impediment to reform (Nguyen et al., 2021). Without sustained, discipline-specific professional development, even willing instructors revert to the familiar transmission model. The solution must be structural: universities serious about transformation must establish well-resourced teaching development centers staffed by experts who work alongside faculty to redesign courses, develop active materials, and refine facilitation skills over multiple semesters—not in isolated workshops.

A second cluster of barriers concerns resources and reward structures. Developing high-fidelity virtual simulations (for an aseptic fill-finish line or multi-column continuous chromatography) demands interdisciplinary teams (bioprocess engineers, software developers, UX designers, assessment specialists) requiring significant, sustained funding seldom available through conventional educational budgets (Radianti et al., 2020). Even once built, maintenance and curricular integration demand ongoing support. Simultaneously, promotion and tenure systems that weigh research grants and high-impact publications far more heavily than educational innovation disincentivize reform. Overcoming these impediments requires coordinated strategies: cross-institutional consortia to share development costs of open educational resources; revised tenure guidelines recognizing the scholarship of teaching and learning as rigorous intellectual inquiry; and career pathways for faculty specializing in pedagogical leadership rather than penalizing them for deviating from research-first norms.

A third barrier concerns scalability and generalizability. The majority of reforms documented in the literature are implemented as pilots, boutique electives, or single-institution initiatives. While such pilots provide valuable proof-of-concept evidence and localized insights, they offer limited guidance for sustained, system-wide adoption across diverse contexts. This “pilot-to-systemic” gap is well-documented in educational change literature. Kopcha’s (2010) Systems Capacity Theory posits that successful scaling requires simultaneous investment in technological infrastructure, pedagogical training, and administrative leadership—a triad rarely fully resourced in pilot phases (Kopcha, 2010). Dede (2006) distinguishes between scaling up (replicating with fidelity) and scaling deep (adapting to local contexts while preserving core principles) (Dede, 2006), which argued the latter is often more sustainable but requires substantially greater investment. In biopharmaceutics education, the absence of robust scaling frameworks means even the most successful pilots (a well-implemented PBL module or a co-supervised industry project) often remain isolated “islands of excellence” rather than catalysts for systemic transformation (Strimel et al., 2026). This is compounded by limited generalizability of single-institution findings, shaped by unique institutional cultures, student demographics, or local partnerships that cannot be easily replicated (Cobian et al., 2026). As Andrews et al. observed, change efforts often focus on individual-level factors while neglecting systemic conditions necessary for sustainable reform (Table 6) (Reinholz et al., 2021).

**Table 6:** Barriers to pedagogical reform in biotechnology pharmaceutics education and proposed mitigation strategies.

Barrier category	Specific challenge	Proposed mitigation strategy	Ref.
Faculty-related	Lack of pedagogical training; research-focused identity	Establish discipline-specific teaching development centers; sustained mentoring	Nguyen et al. (2021)
Resource-related	High cost of VR/AR simulation development	Cross-institutional consortia; open educational resources	Radianti et al. (2020)
Structural/reward	Promotion and tenure undervalue teaching innovation	Revise guidelines to recognize scholarship of teaching and learning	Impey et al. (2025)
Curricular	Fragmented, multidisciplinary rather than interdisciplinary	Design capstone projects integrating bioprocess, regulatory, ethics, business	Mertz and Neiles (2020)
Evidence gap	Lack of longitudinal data on career outcomes; most studies limited to single-course, short-term assessments	Establish an international multi-institutional cohort consortium tracking graduates over 5–10 years using harmonized objective metrics (promotions, regulatory submissions, patents) and validated professional resilience instruments; secure dedicated funding and industry partnerships	Anggraeni et al. (2023)
Technological	Rapid evolution of AI/AR tools outpaces faculty adoption	Intentional, pedagogy-first integration; technology as servant, not substitute	Zawacki-Richter et al. (2019) and Li et al. (2000)
Scalability	Innovations implemented as pilots or electives; limited evidence of system-wide adoption or generalizability across diverse institutional contexts	Multi-institutional consortia; open educational resources; train-the-trainer faculty development; adaptive implementation frameworks allowing contextual reinvention	Strimel et al. (2026), Cobian et al. (2026), Steinert et al. (2016), and Santangelo et al. (2021)
Faculty-related	Lack of pedagogical training; research-focused identity; limited awareness of concrete models for faculty development	Establish discipline-specific teaching development centers; sustained mentoring; adopt evidence-based program models (CIRTL multi-tiered certification, train-the-trainer, tiered cohort mentoring); embed communities of practice	Hill et al. (2019), Pfund et al. (2012), Hill (2023), Ross et al. (2024), Van De Car et al. (2026), and Dominguez et al. (2025)

#### 5.1.2 Exemplars of successful faculty development programs

A growing body of evidence demonstrates that well-designed faculty development programs can successfully enable the transition to active and student-centered pedagogies across diverse institutional contexts. We highlight four peer-reviewed exemplars spanning research universities, community colleges, and international institutions.

The Center for the Integration of Research, Teaching, and Learning (CIRTL) Network (founded 2003, NSF-funded) comprises 43 universities across the U.S. and Canada (Hill et al., 2019; Pfund et al., 2012; Hill, 2023). Its multi-tiered certification (including Associate, Practitioner, and Scholar levels) requires engagement in workshops, teaching-as-research projects, and learning communities. Participants consistently report that CIRTL training was instrumental in their transition to academic careers.

At Arizona State University, an NSF-funded multi-year program engaged 82 engineering faculty from seven disciplines through a train-the-trainer model. Disciplinary leader pairs attended eight biweekly workshops on Bloom’s taxonomy, learning objectives, active learning, and inclusive teaching, then trained 8–15 faculty in their own disciplines. Evaluation revealed a 13% increase in active learning use and a 34% increase in formative feedback practices; all participants would recommend the program, and a sustainable community of practice emerged (Ross et al., 2024).

The Valencia College Mathematics Department (a Hispanic-Serving Institution in Florida), with NSF support, implemented a tiered cohort mentoring model to promote inclusive active learning. Faculty learned IAL theory and techniques, redesigned classrooms, and analyzed disaggregated student outcome data. A structured mentor training program empowered both mentors and mentees, with participants reporting greater openness to trying new techniques (Van De Car et al., 2026).

At a private Chilean university’s School of Engineering, a program aligned with institutional policies employed the World Café methodology to facilitate collective faculty reflection on student-centered learning. Findings highlighted motivated instructors committed to improvement, which are further supported by comprehensive continuing education. The study concluded that successful initiatives hinge on robust faculty-administration collaboration and tailored training (Dominguez et al., 2025).

### 5.2 Strengthening the evidence base: the longitudinal imperative

Additionally, another methodologically profound barrier concerns the temporal scope of the evidence base. As noted in Section 4, the overwhelming majority of studies report outcomes measured at single-course or semester endpoints. Meta-analyses robustly demonstrate immediate gains in examination performance, conceptual understanding, and self-reported engagement following active pedagogies. However, the critical translational question—whether these reforms translate into superior professional performance, accelerated career progression, and sustained innovation capacity over 5–10 years of industrial practice—remains largely unanswered. This lacuna is not unique to biopharmaceutics; comprehensive longitudinal reviews have repeatedly identified the scarcity of long-term graduate tracking as a fundamental limitation in higher education research (Mayhew et al., 2016; Davis, 2006). In STEM and engineering education specifically, the absence of systematic longitudinal cohort studies undermines the credibility and scalability of reform movements (Karen et al., 2025; Chen et al., 2026). To move beyond passive acknowledgment, we propose a concrete, actionable framework for future longitudinal research, which is comprised of five interconnected components (summarized in Tables 7, 8).

**Table 7:** Composition and core functions of the integrated student-centered teaching ecosystem.

System module	Sub-content	Core function in talent training
Active teaching module	PBL, CBL, flipped classroom	Cultivate interdisciplinary thinking and active problem-solving ability
Practical teaching module	Virtual simulation + physical wet laboratory	Improve operational skills, safety awareness and troubleshooting ability
Industry docking module	Internship, joint projects, mini-CMC	Realize school-enterprise docking, enhance career adaptability
Value education module	Ethics and social responsibility embedded teaching	Cultivate professional ethics and comprehensive literacy
Assessment guarantee module	Diversified authentic assessment	Guide students to focus on ability improvement rather than rote memory
Institutional support module	Faculty training, funding, policy incentive	Ensure sustainable implementation of teaching reform

**Table 8:** Framework for future longitudinal studies tracking biopharmaceutics graduates.

Component	Description	Key elements	Ref.
Core research questions	Guiding questions linking pedagogy to long-term outcomes	Career advancement; innovation capacity; professional resilience; component-specific effects	Anggraeni et al. (2023) and Mayhew et al. (2016)
Study design and sampling	Multi-institutional cohort design with longitudinal tracking	5–10 year tracking; biennial data waves; stratified sampling across institution types, demographics, and professional roles	Karen et al. (2025) and Chen et al. (2026)
Indicator system	Triangulated measures from multiple sources	Graduate self-reports (resilience, ethical judgment); employer evaluations (performance rubrics); objective career metrics (promotions, patents, regulatory submissions)	Villarroel et al. (2018) and Freeman et al. (2014)
Implementation roadmap	Phased approach with milestones	Phase 1 (Foundation: Years 1–2); Phase 2 (Data Collection: Years 3–7); Phase 3 (Analysis and Dissemination: Years 8–10)	Borrego and Henderson (2014)
Governance structure	Multi-stakeholder oversight	Steering committee (academia, industry, professional bodies); external advisory board	Reinholz et al. (2021) and Borrego and Henderson (2014)

First, core research questions should address: (1) To what extent do active pedagogical reforms predict career advancement trajectories (e.g., time-to-promotion, leadership roles) compared to traditional instruction? (2) Do graduates of reformed programs demonstrate superior innovation capacity, as measured by patent filings, regulatory submissions, and technology transfer projects? (3) How does professional resilience evolve over time, and what pedagogical factors predict sustained resilience? (4) What is the relative contribution of different pedagogical components (e.g., PBL vs. industry internships) to long-term outcomes?

Second, study design and sampling should adopt a prospective, multi-institutional cohort design with a minimum 5-year tracking period (ideally 10 years) and biennial data collection waves. Stratified sampling should ensure representation across institutional types (research-intensive, teaching-intensive, comprehensive), student demographics, and professional roles (process development, regulatory affairs, quality assurance, manufacturing science, academia). Baseline measures should be collected at graduation.

Third, a triangulated indicator system should integrate three data sources: (a) graduate self-reports measuring professional identity, job satisfaction, self-efficacy, and ethical decision-making using validated instruments; (b) employer evaluations of innovation, teamwork, adaptability, and regulatory compliance using standardized rubrics; and (c) objective career metrics including time-to-first-promotion, regulatory submissions led or co-authored, patent filings, technology transfer projects, and job mobility patterns. This multi-source approach mitigates single-source limitations and provides a comprehensive picture of professional success.

Fourth, an implementation roadmap should proceed in three phases: Phase 1 (Years 1–2): Foundation — establishing the consortium, securing funding, developing instruments, and obtaining ethics approvals; Phase 2 (Years 3–7): Data collection — recruiting cohorts, administering surveys, conducting employer interviews, and tracking objective metrics; Phase 3 (Years 8–10): Analysis and dissemination — conducting longitudinal data analysis (e.g., growth curve modeling, survival analysis), publishing findings, and refining recommendations. Critical resources include dedicated research staff, secure data infrastructure, and formal industry partnerships.

Fifth, a governance structure should comprise a steering committee with representatives from academia, industry, and professional bodies to ensure stakeholder alignment and sustained engagement, and an external advisory board for methodological oversight.

This framework offers a realistic and phased pathway for generating the longitudinal evidence the field urgently needs. While resource-intensive, the investment is justified by the high-stakes nature of biopharmaceutical education, where graduates are entrusted with developing life-saving therapies. We urge funding agencies, academic institutions, and industry partners to prioritize such studies as a collective responsibility.

### 5.3 Future pathways: technology, interdisciplinarity, and human-centric education

Emerging technologies offer pathways to reshape the reach and personalization of biopharmaceutics education. Artificial intelligence (particularly large language models and adaptive learning engines) could enable personalized learning pathways that respond in real time to conceptual misunderstandings, recommending targeted micro-modules before students proceed to design projects (Zawacki-Richter et al., 2019). Augmented reality, overlaid onto physical laboratory equipment, could enable experts to supervise hands-on techniques remotely. Through a shared AR interface, an aseptic technique specialist located in a different facility or country could observe a student’s movements in real time and deliver corrective feedback during gowning or vial manipulation practice (Li et al., 2000). Early iterations of these technologies are already deployed in medical and industrial training. The challenge lies in harnessing them intentionally so that they amplify, rather than replace, the human dimensions of mentorship and judgment at the heart of professional formation.

The complexity of the biopharmaceutical profession also demands a curriculum that is genuinely interdisciplinary, not merely multidisciplinary. The bioprocess engineer of the near future must navigate cell culture dynamics, regulatory logic (ICH Q12), business imperatives (cost-of-goods modeling), and bioethical implications (cell and gene therapy access). Modules that merge bioprocess engineering with regulatory science, business strategy, and bioethics, requiring students to integrate these perspectives in a capstone design project, will better reflect professional decision-making and prepare graduates for leadership at the intersection of science, commerce, and public trust (Mertz and Neiles, 2020).

### 5.4 Strategies for scaling and generalization

Despite these challenges, actionable strategies exist for scaling pedagogical reforms across diverse institutional contexts. We propose four complementary approaches:

First, multi-institutional consortia distribute costs and risks while generating evidence of generalizability. By implementing common interventions across institutions with varying sizes, resources, and student populations, consortia disentangle program effects from local contextual factors, producing more robust and transferable findings. The European PBL Network and the US-based CIRTL provide instructive precedents in STEM education (Borrego and Henderson, 2014).

Second, open educational resources (OERs, including adaptable PBL case libraries, virtual simulation modules, and assessment rubrics) lower adoption barriers for resource-constrained institutions. Meta-analyses confirm that OERs deliver comparable learning outcomes to proprietary materials while reducing costs and increasing faculty willingness to experiment (Hilton, 2016).

Third, train-the-trainer faculty development models build pedagogical capacity sustainably. Rather than relying on external experts, these models equip “master instructors” within each institution to mentor colleagues, which is capable of creating self-sustaining professional growth. Systematic reviews confirm that train-the-trainer models produce durable changes in teaching practice when combined with ongoing peer observation and feedback (Steinert et al., 2016).

Fourth, adaptive implementation frameworks recognize that no single approach fits all contexts; effective scaling requires flexibility to adapt core principles locally—what Rogers (1995) terms “reinvention.” A PBL module designed for a well-resourced research university may need reconfiguration for a teaching-intensive institution with larger classes and fewer teaching assistants (Miao et al., 2024). Cross-institutional comparisons reveal that PBL implementation is shaped by local conditions, and these include tutor availability, student preparation, and institutional support (Musau et al., 2026). The adaptive fidelity framework offers a model that balances fidelity to core principles with teacher-led adaptation responsive to contextual moderators such as resources and student readiness (Estacio et al., 2025).

These strategies suggest that scaling is not a linear “roll-out” but an iterative, context-sensitive process of mutual learning between pilot and adopting institutions. For biopharmaceutics educators, this implies a shift from seeking a single “best practice” to cultivating a community of practice that shares failures, adaptations, and contextual insights alongside successes. The strategies are summarized in Table 9.

**Table 9:** Scaling strategies for pedagogical innovations.

Barrier to scaling	Corresponding scaling strategy	Mechanism of action	Ref.
High development costs (VR/AR, PBL cases)	Multi-institutional consortia; open educational resources (OERs)	Distribute development costs; enable reuse and adaptation across institutions	Radianti et al. (2020) and Hilton (2016)
Limited faculty pedagogical training	Train-the-trainer faculty development models	Build local capacity through peer mentoring and sustained professional learning communities	Nguyen et al. (2021) and Steinert et al. (2016)
Contextual mismatches (different student demographics, resources, institutional cultures)	Adaptive implementation frameworks; “reinvention” with fidelity to core principles	Preserve pedagogical integrity while accommodating local variations	Miao et al. (2024) and Musau et al. (2026)
Insufficient evidence of generalizability	Multi-institutional cohort studies with harmonized metrics	Generate transferable evidence; disentangle program effects from context	Strimel et al. (2026) and Cobian et al. (2026)
Institutional silos; lack of cross-departmental coordination	Community of practice models; cross-institutional networks	Create platforms for shared learning; normalize adaptation over replication	Borrego and Henderson (2014) and Santangelo et al. (2021)
Misalignment with existing curricular structures	Phased implementation; modular integration	Introduce innovations incrementally; allow co-existence with traditional methods	Jackson (2013) and Miao et al. (2024)

## 6 Conclusion

The pedagogical landscape of biotechnology pharmaceutics education stands at a critical juncture. The rapid scientific and industrial advances that have reshaped the biopharmaceutical sector (such as monoclonal antibodies, cell and gene therapies, continuous manufacturing, and precision medicine) have rendered the traditional lecture-dominated, fragmented, and passive instructional model not merely inadequate but actively counterproductive. This narrative review has surveyed the principal reform movements that have emerged in response: problem-based and case-based learning, flipped and blended classrooms, virtual simulation and digital laboratories, industry–academia integration, and curriculum-embedded ethical education. Each of these strategies, when thoughtfully implemented, has demonstrated measurable benefits in cultivating the interdisciplinary reasoning, practical problem-solving, and professional judgement that define competent biopharmaceutical scientists.

Yet the central argument of this review is not that any single innovation represents a panacea, but rather that an integrated, student-centered ecosystem—one that combines multiple active pedagogies, authentic experiential learning, robust digital tools, and aligned assessment—constitutes the most promising path forward. A standalone case study or an occasional virtual lab exercise, however well designed, cannot compensate for a curriculum that otherwise rewards passive memorization. True transformation requires coherence: PBL triggers that are reinforced by flipped-classroom preparation, virtual simulations that feed into industry-mentored projects, and ethics discussions that permeate technical modules rather than residing in a separate, marginalized course. Moreover, the transition from isolated pilots to systemic reform demands more than institutional will; it requires structured strategies for scaling and generalization. We have therefore provided a practical framework, drawing on multi-institutional consortia, open educational resources, train-the-trainer models, and adaptive implementation, to guide educators and administrators in translating local successes into sustainable, context-sensitive institutional change. This framework, while preliminary, offers a roadmap for moving beyond the “islands of excellence” that currently characterize much of the reform landscape. Moreover, this ecosystem must be supported by assessment architectures that value process, collaboration, and communication alongside factual recall.

The evidence base, while increasingly robust for short- to medium-term cognitive, motivational, and performance outcomes, remains critically incomplete in its temporal scope. Most studies report gains measured within a single course or semester; robust longitudinal data tracking graduates’ career trajectories, innovation capacity, and professional resilience over 5–10 years remain exceptionally scarce. We have therefore proposed a concrete, actionable framework (an international cohort consortium) to transform this acknowledged limitation into a funded research agenda. This framework-encompassing core research questions, study design, triangulated indicators, a phased implementation roadmap, and a multi-stakeholder governance structure-offers a realistic pathway for generating the evidence needed to validate and refine pedagogical reforms.

Until such data materialize, our recommendations are best understood as evidence-informed projections grounded in sound pedagogical theory and robust short-term findings, rather than definitive claims of lifelong impact. This honest appraisal, however, does not diminish the urgency of reform; it sharpens it by charting the precise empirical course the field must now navigate. Furthermore, the gap between successful pilots and systemic adoption is wide, obstructed by faculty development deficits, resource constraints, and promotion systems that undervalue pedagogical excellence. Addressing these structural barriers is as essential as refining the pedagogies themselves.

Looking forward, emerging technologies (including the artificial intelligence for personalized learning pathways, augmented and virtual reality for immersive procedural training) promise to extend the reach and fidelity of experiential education. Yet technology must remain a servant, not a substitute, for the human elements of mentorship, ethical deliberation, and professional identity formation. The ultimate goal is not merely to produce graduates who can execute protocols or pass examinations, but to cultivate practitioners who can navigate ambiguity, integrate knowledge across boundaries, and act with integrity when the path is not pre-scribed. That aspiration, at once ambitious and essential, is the true measure of pedagogical reform in biotechnology pharmaceutics education.
